# Early Treatment of Acute Stage 0/1 Diabetic Charcot Foot Can Avoid Major Amputations at One Year

**DOI:** 10.3390/jcm13061633

**Published:** 2024-03-13

**Authors:** Cristina Bittante, Valerio Cerasari, Ermanno Bellizzi, Raju Ahluwalia, Michela Di Venanzio, Laura Giurato, Aikaterini Andreadi, Alfonso Bellia, Luigi Uccioli, Davide Lauro, Marco Meloni

**Affiliations:** 1 Division of Internal Medicine, Mater Salutis Hospital Legnago, 37045 Legnago, Italy; 2 Ortopedia CORM Srl, 00198 Rome, Italy; 3 Division of Endocrinology and Diabetology, Department of Medical Sciences, Fondazione Policlinico “Tor Vergata”, 00133 Rome, Italy; 4 Department of Orthopaedics, King’s College Hospital, London & King’s College London & Royal College of Surgeons of England, London WC2A 3PE, UK; 5 UOSD Diabetologia San Camillo De Lellis Hospital, 02100 Rieti, Italy; 6 CTO Andrea Alesini Hospital, Division of Endocrinology and Diabetes, Department of Systems Medicine, University of Rome Tor Vergata, 00145 Rome, Italy

**Keywords:** diabetes, Charcot neuro-osteoartropathy, neuropathy, offloading, amputation

## Abstract

**Background:** If unrecognized, Charcot neuro-osteoarthropathy (CNO) can be a devastating complication of diabetes. **Methods:** The aim of this retrospective study was to evaluate the outcomes in a cohort of diabetic patients diagnosed with active CNO managed in a tertiary level diabetic foot clinic (DFC). We included consecutive patients with active CNO, stage 0–1, according to the Eichenholtz–Shibata classification, who were referred from 1 January 2019 to 27 September 2022. Diagnosis of CNO was based on clinical signs and imaging (X-rays and magnetic resonance). All patients were completely offloaded by a total-contact cast (TCC) or removable knee-high device. Each patient was closely monitored monthly until CNO remission or another outcome. At 12 months of follow-up, the following outcomes were analyzed: remission, time to remission, major amputations (any above the ankle), and surgical indication. **Results:** Forty-three patients were included. The mean age was 57.6 ± 10.8 years; 65% were males and 88.4% had type 2 diabetes, with a mean duration of 20.6 ± 9.9 years. At baseline, 32.6% was affected by peripheral artery disease. Complete remission was recorded in 40/43 patients (93%), with a mean time to remission of 5.6 ± 1.5 months; major amputation and surgical indication occurred, respectively in 1/43 patients (2.3%) and 3/43 patients (7%). **Conclusions:** Early treatment of active Stage 0/1 CNO leads to high rates of remission and limb salvage.

## 1. Introduction

If unrecognized, Charcot neuro-osteoarthropathy (CNO) can be a destructive and devastating complication of neuropathy in patients affected by diabetes, as it can lead to joint and bone deformities, development of ulcers and, ultimately, to amputation [1,2,3].

CNO has historically been considered a rare complication of diabetes, but recent studies suggest that the prevalence of CNO is substantially underestimated [4]. A novel analysis by Lazzarini et al. underlined that 58% of the total global disease burden caused by Diabetic Foot Disease is the disability burden: this burden for the most part is at the expense of people with neuropathy without ulcers (thus including CNO) [5]. A nationwide cohort study conducted in Sweden showed the increasing prevalence of diabetic CNO from 0.55 to 0.79% (from 1.06 to 1.97% for type 1 diabetes) during the period 2006–2016 [6]. In addition, Svendsen et al. showed that the prevalence of Charcot foot was 0.56% (from 1995 to 2018) in a very large cohort of patients included in this survey (i.e. The Danish National Patient Register) [4].

In recent years, many authors have provided evidence-based guidelines for the management of CNO [3,7], supporting clinicians in the diagnosis and treatment of acute and chronic stages. Moreover, in 2023 the International Working Group on Diabetes Foot (IWGDF) has released its first own guidelines on the diagnosis and treatment of active diabetic CNO [8]. These specific guidelines recommend the prompt initiation of knee-high immobilization/offloading in case of suspected active CNO, while other tests are conducted to confirm or rule out CNO diagnosis. A non-removable knee-high device (total contact cast, TCC, or a device rendered non-removable as second choice) is considered the gold standard for treatment, whilst a removable knee-high device is considered a third-choice option.

However, it is important to point out that these guidelines were developed for CNO and intact skin. It is not uncommon that patients with active CNO and diabetes also present with an active infected DFU or other absolute/relative contraindications to the use of a TCC/non-removable knee high device, such as osteomyelitis or peripheral artery disease (PAD) [9]. Moreover, the application and monitoring of a TCC needs a certain level of expertise, not available everywhere, and the TCC can itself cause blistering or new lesions, in addition to worsening the risk of falls [10,11,12].

Today, it is recognized that early detection, immobilization and reduced weight-bearing on the affected limb are effective in reducing the development of deformities [13,14].

The new IWGDF guidelines indicate the need for further investigating which offloading modality is the most effective in terms of remission rates, while simultaneously taking into account patients’ acceptance and adherence, socio-economic factors and cost-effectiveness.

Nonetheless, to date the studies evaluating the outcomes of patients affected by active CNO are not fully comparable since they differ in inclusion/exclusion criteria and the definition of the outcomes is poorly standardized (i.e., definition of remission); also, treatment protocols can be highly variable from one center to another due to available resources and expertise. CNO is still considered nowadays a terrifying disease which can lead to deformities, ulceration, poor quality of life and, ultimately, amputation. However, from our clinical experience, an early diagnosis combined with an immediate appropriate treatment and a close follow-up seems to allow for favorable outcomes.

CNO has been always considered a deadly issue for clinicians dedicated to diabetic foot care, and several studies have been developed to evaluate outcomes of patients with active diabetic CNO [15,16,17,18,19,20]; based on this statement, the authors thought to analyze what happens in daily clinical practice to better understand the course of active CNO, and in order to improve its treatment also in our specific reality.

Accordingly, the aim for this study was to evaluate the outcomes of the early treatment of active Stage 0/1 CNO in diabetic patients managed in a specialized DFC.

## 2. Materials and Methods

### 2.1. Patients

This retrospective observational cohort study analyzed consecutive diabetic patients diagnosed with an active CNO stage 0 or 1 according to the Eichenholtz–Shibata classification [21].

Patients included were enrolled from 1 January 2019 to 27 September 2022; they were managed in a specialized tertiary-level diabetic foot clinic (Department of Endocrinology and Diabetology, at the University Hospital of Rome Tor Vergata).

Exclusion criteria were the absence of diabetes, chronic stages of CNO, and the presence at the first assessment of extensive soft tissue loss due to soft tissue or bone infection, bone fractures or joint deformities not allowing a potential surgical limb salvage/reconstruction (Table 1). The feasibility of potential surgical limb salvage/reconstruction was made by diabetic foot surgeons with a long clinical and surgical experience on diabetic foot in association with diabetic foot surgeons specifically dedicated to the reconstruction of Charcot foot.

Overall, 80 patients with CNO were considered for the study; 34 patients with chronic CNO were excluded from the study according to the aim and inclusion criteria. Out of the 46 patients with Stage 0/1 CNO initially retrospectively included, 3 were excluded from the final data set. A total of 2 patients were excluded after the first clinical assessment due to an extensive tissue loss not suitable for the conservative therapy (destructive osteomyelitis with severe ankle deformity/instability); the third patient was excluded from the final data set since no regular follow-up was available. Hence, forty-three episodes (from now on, “patients”) of active CNO were included. Six patients experienced a non-simultaneous bilateral involvement. Two patients recalled a previous episode of contralateral active CNO (before their first access to our DFC): no data are available regarding these two episodes.

Data were collected in a local database and retrospectively analyzed. Baseline demographic, clinical and ulcer findings were recorded.

The study has been conducted and approved according to local ethics committee policy. At admission, patients provided their consent.

### 2.2. Diagnosis of CNO

Active CNO was suspected in case of an increase in temperature (>2 °C), oedema, with a combination of redness and rarely pain, compared to the contralateral foot, in patients with a documented diagnosis of diabetes and neuropathy. The clinical suspicion was then confirmed by performing radiological investigations such as plain X-rays and specifically magnetic resonance imaging (MRI).

Stage 0 was defined in case of clinical signs, normal X-rays but evidence of osseous abnormalities at MRI (such as bone oedema and acute fragmentations) [21].

Stage 1 was defined as the presence of clinical signs and bone alterations at imaging (MRI and/or X-ray), with the maintenance of the plantigrade foot and the ankle joint stability, and a preserved deambulation.

### 2.3. Other Variables of Interest and Treatment Protocol

Demographic and clinical data were collected for each patient from the time of diagnostic suspicion. In particular, information on age, gender, type of diabetes, duration of diabetes, HbA1c, chronic complications of diabetes and comorbidities such as neuropathy, peripheral artery disease, arterial hypertension, ischemic heart disease, dyslipidemia and end-stage renal disease (ESRD) were obtained.

Peripheral neuropathy was considered in case of loss of protective sensation detected through vibration perception (128 Hz tuning fork) or Semmes–Weinstein 10 g monofilament [22,23]. The presence of small-fiber neuropathy was not investigated, even if in this cohort all patients with suspected CNO tested positive for monofilament and vibration tests. Nephropathy was considered in the case of micro- (30–300 mcg/mg creatinine) or macro-albuminuria (>300 mcg/mg creatinine) [24].

Coronary heart disease (IHD) was considered in the case of previous acute coronary syndrome or coronary revascularization, evidence of angina, or significant changes in electrocardiography (above or under-leveling ST, q wave, inversion of T wave, and new left bundle branch block). Cerebrovascular disease was considered in the case of previous cerebrovascular ischemia, previous carotid revascularization, or significant carotid artery disease (occlusion/stenosis > 70%) [24].

Arterial hypertension was defined as blood pressure persistently > 130/80 mmHg and/or the current use of anti-hypertensive medications; hypercholesterolemia was defined as low density lipoproteins (LDL) > 70 mg/dL or the need for lipid-lowering therapy.

End-stage renal disease (ESRD) requiring dialysis was considered in the case of chronic renal replacement therapy [24].

The presence of PAD was evaluated for all patients, and it was defined as absence of palpable distal pedal pulses, or TcPO2 < 50 mmHg and/or stenosis/occlusions at duplex-US examination. Lower limb revascularization was performed in case of critical limb ischemia (TcPO2 < 30 mmHg or pathologic duplex-US) and concomitant active diabetic foot ulcer (DFU).

### 2.4. Management of Active CNO

All patients received knee-high offloading (TCC or knee-high removable device) in addition to complete immobilization: patients were taught to use a wheelchair at all times, thus further reducing weight-bearing activity. Moreover, ulcer location/size, infection and ischemia issues were treated according to the standard of care [25,26,27,28]. Our offloading regimen is based on our local protocol; even though not all the guidelines/best practice statements recommend a complete immobilization, we preferred a forced immobilization in order to avoid the risk for further dislocations, fractures and deformities, especially in Stage 1 patients.

Patients without ulcers nor PAD were treated by TCC; patients with active non infected DFU without PAD were treated with a TCC or knee-high removable device based on clinical assessment. Patients with an infected ulcer (with or without PAD) and patients with PAD were prescribed a knee-high removable device. For all cases, absolute offloading was recommended until active CNO remission.

The clinical activity of CNO was monitored every 3–6 weeks until remission or definitive outcome. At each visit, a close clinical evaluation was performed, including the re-assessment of acute signs (oedema and hyperemia), the presence of new deformities, the evolution of previous deformities, and the integrity and physiological mobility of the ankle joint. In addition, serial temperature measurements of the affected limb compared to the contralateral were performed. For the measurements of foot temperature infrared thermometry was used. Temperature measurements were performed after an adequate time of acclimatization (fifteen minutes). The medical device we used was Termoskin 2.1–12 V by Meteda (via Silvio Pellico 4, san Benedetto del tronto, Ascoli Piceno, Italy).

The frequency of each visit and measurements were established according to the fluctuation of clinical oedema, the offloading device (i.e., higher for TCCs, lower for removable devices), the CNO location (i.e., higher for the ankle, lower for the forefoot), the presence of DFU, and PAD. This evaluation was performed according to a clinical assessment and current IWGDF guidelines.

Once the remission of active CNO was achieved, patients received a custom-made orthotic shoe, with a rigid outsole and custom-made insoles, with the aim to accommodate any deformity and to redistribute plantar pressure, in order to avoid ulcerations and to guarantee back-to-walking.

### 2.5. Outcomes

The main outcome of this study was to determine the rate of remission of active CNO and time to remission during the 12 months follow-up period. The remission was defined as the normalization of temperature difference between the affected and contralateral limb, measured in two consecutive ambulatory visits 4 weeks apart [29,30], with the absence of new significant deformities suitable for surgical intervention/surgical reconstruction. Only in cases of persistent clinical signs and/or temperature difference > 2 °C, MRI was performed to revise, discuss and modify (if required) the initial clinical assessment and management; however, we did not use MRI alone to determine the clinical remission. As has been well reported, the remission documented by MRI can be achieved even only 3–6 months after clinical healing [31], while in the current study the aim of the authors was to consider remission using the clinical evaluation.

Other outcomes of interest included the rate of major amputation (defined as any amputation above the ankle), mortality, and surgical indication due to the development of deformities.

### 2.6. Statistical Analysis

Continuous variables are expressed as mean ± standard deviation, while categorical variables are presented as percentage or proportion.

For patients presenting with bilateral involvement, each episode of active CNO was considered separately. In order to analyze the localization according to the Sanders–Frykberg classification, in case of multiple simultaneous localizations we decided to evaluate each pattern separately.

Statistical analysis was performed with STATA 14.2.

## 3. Results

### 3.1. Patients

The mean age was 57.6 years (SD 10.8 years), with a majority of male (65.1%) and Type 2 Diabetes Mellitus (T2DM) (88.4%) patients. The mean duration of diabetes was 20.6 years (SD 9.9 years). Over one third of patients had PAD, while only one was undergoing hemodialysis for ESRD. Almost 3 out of 4 were affected by arterial hypertension, a lower proportion had dyslipidemia or coronary heart disease. At baseline 16.3% had a concomitant active DFU, while 11.6% had an infected DFU (Table 2). According to the Eichenholtz–Shibata classification, 16/43 (37.2%) patients were classified as Stage 0, and 27/43 (62.8%) patients were classified as Stage 1.

Many patients presented simultaneous multiple patterns of localization (twenty-eight out of forty-three patients). The vast majority of patients experienced midfoot involvement (Pattern II–III) (Table 3).

### 3.2. Outcomes

The overall remission of active CNO was reached in 40 out of 43 patients, with a 93.0% remission rate at 12 months. Remission rate was 100% in Stage 0 patients and 88.9% in Stage 1 patients (*p* = 0.03). The mean time to remission for the whole population was 5.6 ± 1.5 months, while the time to remission for Stage 0 was 3.6 ± 0.9 months and for Stage 1 6.7 ± 1.8 months (*p* = 0.01; Table 4).

In this sample, the rate of major amputation at 12 months was 2.3% (1/43 patients); due to severe ankle/foot deformity, ulceration and severe infection were not suitable for limb salvage. The rate of mortality was 2.3% as well. Amputation and deaths were recorded in both cases among the Stage 1 group.

The overall rate of new deformities requiring surgical indication was 7.0% (3/43 patients); this evaluation included also the only patient who underwent major amputation. No patients belonging to the Stage 0 subgroup developed deformities requiring surgical correction during the follow-up period, while surgical reconstruction was required in 3/27 patients belonging to the Stage 1 group (0% vs. 11.1%, *p* = 0.03, respectively).

## 4. Discussion

Our findings showed that early detection and rigorous treatment of active Stage 0/1 CNO leads to high rates of remission and limb salvage, with a complete remission in 93% of cases at 1 year of follow-up. Patients diagnosed at Stage 1 showed worse outcomes in terms of remission rate, longer remission time and a higher rate of new deformities requiring surgical intervention.

Chantelau has described how patients diagnosed at Stage 0 rarely developed deformities when compared to patients receiving a diagnosis of active CNO at Stage 1. In particular, 100% of patients at Stage 1 vs. 9% of patients at Stage 0 progressed to fractures, but the rate of gross deformity followed a similar pattern (respectively, 92.3% vs. 9%) [13]. In our cohort, no patients belonging to the Stage 0 group developed significant deformities at one year follow-up, while the same was recorded in 3/27 (11.1%) patients belonging to the Stage 1 group. Moreover, Wukich and Armstrong in 2011, in a multi-center study, demonstrated that the outcomes of CNO diagnosed at stage 0 depended on the appropriate recognition and early treatment [14]. In fact, 66.7% of patients who suffered a delay in the diagnosis of CNO required surgical treatment, with an average of 2.9 surgeries per limb; on the other hand, only one patient (14.3%) among those with an early recognition developed a midfoot ulceration as a complication of TCC, with a need for debridement and reconstructive surgery. Pakarinen et al., in a small series, showed that the correct diagnosis within 3 months resulted in better functional outcomes and walking distance [16].

Waibel et al., in 2021, analyzed a cohort of 184 Charcot feet including various neuropathy etiologies such as diabetes, ethylism, spinal cord injury, idiopathic and other minor causes. In this cohort managed in a specialized Orthopedics clinic, conservative treatment allowed limb preservation in 93% of cases at 5 years follow-up. The observations included any Eichenholtz–Shibata stage at presentation, and the median casting time was 191 days [32].

In the current retrospective study, the mean remission time was 5.6 months. The mean remission time seems more variable than in previous studies. Armstrong et al., suggested the immobilization time was about 18.5 ± 10.6 weeks, with a back-to-walk time of 28.3 ± 14.5 weeks [33]. All patients were treated initially by TCC, and afterwards a gradual weight bearing with a walker was allowed. An Australian work conducted on 27 patients at stage 0–1 treated by TCC described a median casting time of 4.3 months [34]. The results reflect more or less our data. Nonetheless, some literature data have been reported that CNO may have longer times of activity in comparison to our data or those mentioned above; Gratwohl et al. documented that the range of TCC treatment was 7–640 days in the respect of the CNO active phase [32]. In a similar study which included a cohort of patients with acute diabetic CNO managed during the period 2006–2011, patients were treated by a standard offloading protocol for an average time of 15.12 ± 5.45 months; in this specific study, the remission was considered with the resolution of inflammatory indexes by using PET/CT and MRI imaging [35].

In our cohort, we recorded a major amputation rate of 2.3%. In 2010, Sohn et al. conducted a retrospective analysis on lower limb amputation during a 5-year follow-up in patients affected by CNO or DFU: the amputation rate was, respectively, 4.1 and 4.7 per 100/people-year [36]. The authors underlined how the amputation risk was 12× higher in patients who were affected by both conditions; thus, the ulcer prevention represented a turning point in limb salvage in CNO [37]. In another cohort, the rate of major amputation was relatively low (2.7%) [20] and comparable to the rate observed in our study population. A Danish cohort of 173 diabetic patients affected by CNO treated in a specialized center between 1996 and 2015 reported an approximate major amputation rate of 10% in a median follow-up of 31 months [19]. Another small Norwegian cohort reported a major amputation rate of approximately 15%, for the most part due to hindfoot CNO involvement [15]. The authors are aware that our study provides a rather short follow-up period (12 months); recently, Waibel et al. showed how amputation-free survival rates decrease over the follow-up period, especially for diabetic patients (and dramatically for type 1 diabetic patients) [38]. Therefore, we cannot completely exclude that a longer follow-up period may increasethe rate of major amputation that we have recorded during this year of follow-up.

In our cohort, 7% of patients (3/43) developed deformities that required a surgical approach (all belonging to the Stage 1 subgroup, 11.1%). All of them showed midfoot involvement, and two of them had also an ankle joint involvement; these data suggest the need for a closer follow-up in the case of midfoot/ankle CNO.

In the series by Armstrong et al. almost 25% of patients developed deformities with indication to surgical correction [33]; However, there are no data available on the stage at presentation according to Eichenholtz–Shibata.

In another retrospective analysis by Fabrin conducted in 2000, the rate of patients requiring a surgical approach was less than 5% [17], close to what we observed. As already reported above, our study provided a shorter follow-up period (12 months) than the previous studies, so it cannot be excluded that a longer follow-up period may increase the risk and the rate of severe deformities requiring a surgical reconstruction.

A standardized definition of clinical or radiological remission is to date lacking; also there is no agreement on which diagnostic test can be considered as “gold standard” to ascertain active CNO remission [8,39]. In our study, we used the assessment of the cutaneous temperature difference (<2 °C) in combination with the resolution of the clinical signs (oedema, redness and/or pain if present), a method well accepted by experts [40]. The authors well know that data regarding the effectiveness of temperature measurements to monitor the CNO activity are still uncertain, and previous studies have also evaluated different cut-off points to define the potential remission. Accordingly, authors agree that, for evaluating the remission of CNO, it is appropriate and mandatory to consider several items including temperature measurements, clinical framework and imaging when required.

In 2011 Zampa et al. suggested the use of dynamic MRI at initial evaluation (to confirm the diagnosis), at 3 months (to verify the initial response to treatment) and once clinical stability was reached, in order to confirm the remission [31].

A recent pilot trail has investigated the feasibility of serial MRI every 3 months to adjuvate the identification of CNO remission, defined as the absence of subchondral marrow oedema [41]. The outcomes of this study would justify a randomized clinic trail with the aim of defining the usefulness of MRI in identifying remission, so that immobilization times can be contained. The main limitations to the use of MRI are indeed the high costs and the possibility to access this exam with the proper timing; also, bone oedema can lead to overestimation of activation [31].

In our study, the majority of patients (approximately 90%) was treated by a knee-high removable device. The proportion of patients treated by removable knee-high device is usually less represented (for example, 42% in Zampa et al. [31] and 31% in Petrova et al. [42]). Treatment for active CNO is represented by knee-high immobilization/offloading and it should be started promptly at clinical suspicion. TCC is the gold standard device, while non removable or removable knee-high devices are considered, respectively, the second and third choice. However, the IWGDF 2023 guidelines focus on CNO with intact skin: often patients at first visit may present absolute/relative contraindications to the TCC/non removable device, such as PAD (in our cohort > 1/3), the concomitant presence of infected DFU or a high risk of falling. Moreover, not every clinic has the expertise, skills or resources to provide the correct application and management of a TCC and its potential harmful complications.

The same IWGDF 2023 guidelines underline the need for further investigation in order to identify which offloading modality is the most efficacious in terms of remission, while taking simultaneously in account patients’ acceptance, adherence, socio-economical factor and cost-efficacy [8].

Our offloading regimen is based on our local protocol and is not so far from the recommendations used by the IWGDF; we preferred to reinforce immobilization in order to avoid the risk of potential deformities, especially in Stage 1 patients; the usual absence of pain in diabetic people with active CNO may increase the risk that patients continue to walk regularly, resulting on repetitive trauma. Two studies, by Dodd and Weibel, respectively, [3,7] have reported good outcomes in terms of remission, time of remission and safety adopting a less rigorous protocol (protected weight-bearing by using an irremovable device) in comparison to that adopted in our current study [3,7]. On the other side, it has been also reported that an early and less rigid protocol has been associated with an increased risk of recurrence [18]. Therefore, even if authors are used to apply a rigorous non weight-bearing protocol, they are aware that the effectiveness of an absolute immobilization is uncertain and any protocol should be adapted based on each specific case in the respect of evidence based studies and guidelines recommendations.

Besides the offloading device used, what clearly arises is the effectiveness of an early diagnosis and early offloading in reducing the development of deformities. In fact, both the duration and the intensity of weight-bearing activity are associated with the development of deformities in active CNO [43].

Despite the evidence from the literature, a recent metanalysis underlined how to date over 50% experience a delay in diagnosis estimated in 87 days, contributing to consequent worse patient outcomes (namely, major amputations and development of deformities/joint instability requiring surgical correction) [44].

To date, the role of surgery in active CNO has been defined quite clearly; primary indications include recurrent ulceration (suggestive of unstable foot), substantial axial malalignment, deep infection, and pain [45]. Pinzur et al. pointed out that the current goals of surgical treatment are the resolution of infection, limb salvage, and the restoration of a plantigrade “shoeable” foot [46]. In specific cases, surgical reconstruction should be considered a limb salvage procedure and a disability recovery procedure. Nonetheless, CNO surgery requires a long expertise and often a multidisciplinary management in the respect of concomitant comorbidities affecting diabetic foot patients.

In the recent years, surgical reconstruction of Charcot foot is not only addressed to some extreme case of deformity and instability, but also for active and deformed CNO with the aim of restoring anatomy and preventing further deformities, ulceration and amputation [5].

The authors believe that the strength of the current study can be found in the good outcomes in terms of avoiding major amputations/surgical interventions and in terms of remission at one year in those patients with Stage 0–1 without the presence of severe deformities and foot instability at the initial assessment. A prompt approach and a close follow-up seem also to allow a short time for clinical remission. However, the rigorous offloading we have applied has required a complete immobilization, thus potentially worsening the quality of life during the time of treatment. Our results were favorable even with the use of a removable device, prescribed as protection and associated with complete immobilization and absolute offloading. The choice of such removable device permits to substitute the TCC, which often is not applicable/available and carries high iatrogenic risks (besides more frequent ambulatory visits and strong limitation to the patient).

Our study has some important limitations to highlight.

First, it was a retrospective monocentric study where no control group was identified. Our sample was small but relatively acceptable if we consider that usually in CNO studies the sample is rarely much larger.

Another important limitation may be considered the use of clinical signs and temperature difference for assessing remission without the support of imaging, even though the same was the aim of the authors. The presence of small-fiber neuropathy was not investigated, and even though all patients included tested positive for monofilament and vibration tests, we are aware that not performing a complete neurologic investigation comprehensive of small fiber analysis might miss some cases of CNO in daily clinical practice. The short follow-up period, in comparison to some similar studies on CNO, may underestimate the rate of long-term complications (i.e., major amputations, development of deformities requiring a surgical approach). Finally, our results cannot be generalized to the non-diabetic population, since we only investigated diabetic CNO.

Our future perspectives would be to extend the follow-up duration in order to identify long-term complications, to analyze the rate of development of new DFUs, to analyze the remission times in relation to localization (Sanders and Frykberg classification) and to analyze the outcomes in relation to the type of offloading device used and the presence of PAD.

## 5. Conclusions

In conclusion, our study provides further evidence on how active Stage 0/1 CNO can be a disease with a high rate of remission and a low rate of major amputations at one year, if promptly recognized and treated at the earlier stages.

## Figures and Tables

**Table 1:** Summary of inclusion and exclusion criteria.

Inclusion Criteria	Exclusion Criteria
Diabetes mellitus	Absence of diabetes mellitus
Peripheral neuropathy	Chronic stages of CNO ^1^ (inactive CNO)
Active CNO, E-S ^2^ Stage 0 or 1	Presence at first assessment of extensive soft tissue loss, infection, bone fractures or joint deformities not allowing a potential surgical limb salvage/reconstruction
Presence or absence of DFU ^3^	Lost to follow-up
	Advanced stages of active CNO (≥2 according to E-S)

^1^ CNO: Charcot neuro-osteoarthropathy; ^2^ E-S: Eichenholtz–Shibata classification; ^3^ DFU: diabetic foot ulcer.

**Table 2:** Patients’ characteristics at baseline.

Variables	Values (N = 43)
Age (years)	57.6 ± 10.8
Gender (M, %)	28/43 (65.1)
Type 2 Diabetes Mellitus (%)	38/43 (88.4)
Duration of diabetes (years)	20.6 ± 9.9
HbA1c (%)	8.3 ± 2.6
Peripheral artery disease (%)	14/43 (32.6)
Coronary heart disease (%)	7/43 (16.3)
Dialysis (%)	1/43 (2.3)
Arterial Hypertension (%)	31/43 (72.1)
Dyslipidemia (%)	16/43 (37.2)
Active Diabetic Foot Ulcer (%)	7/43 (16.3)
Active Infected Foot Ulcer (%)	5/43 (11.6)
Stage 0 (%)	16/43 (37.2)

Continuous variables expressed as mean ± standard deviation (SD); categorical variables expressed as proportion (%).

**Table 3:** Frequency of Sanders–Frykberg’s patterns of localization.

Sanders–Frykberg Classification	Patients (n.)	%
I	6/43	14.0
I–II	1/43	2.3
II	8/43	18.6
II–III	19/43	44.2
III–IV	5/43	11.7
IV	1/43	2.3
II–III–IV–V	1/43	2.3
III–IV–V	1/43	2.3
IV–V	1/43	2.3

**Table 4:** Summary of outcomes of interest at 12 months.

Outcome	Total (n = 43)	Stage 0 (n = 16)	Stage 1 (n = 27)	*p*
Remission rate: n (%)	40/43 (93.0)	16/16 (100)	24/27 (88.9)	**0.03**
Time to remission (months): mean (SD ^1^)	5.6 (1.5)	3.6 (0.9)	6.7 (1.8)	**0.01**
Major amputation rate: n (%)	1/43 (2.3)	0/16 (0)	1/27 (3.7)	0.6
Mortality rate: n (%)	1/43 (2.3)	0/16 (0)	1/27 (3.7)	0.6
Rate of development of deformities requiring surgical intervention: n (%)	3/43 (7.0)	0/16 (0)	3/27 (11.1)	**0.03**

^1^ SD: standard deviation.

## Data Availability

The raw data supporting the conclusions of this article will be made available by the authors on request.
